# Advancing vaccine-based immunotherapy in glioblastoma treatment

**DOI:** 10.1093/noajnl/vdaf135

**Published:** 2025-06-24

**Authors:** Desh Deepak Singh, Shafiul Haque, Abhishek Kumar Singh, Dharmendra Kumar Yadav

**Affiliations:** Amity Institute of Biotechnology, Amity University Rajasthan, Jaipur, India; Department of Nursing, College of Nursing and Health Sciences, Jazan University, Jazan, Saudi Arabia and School of Medicine, Universidad Espiritu Santo, Samborondon, Ecuador; Manipal Centre for Biotherapeutics Research (MCBR), Manipal Centre for Biotherapeutics Research, Manipal Academy of Higher Education, Manipal, India; Department of Biologics, College of Pharmacy, Gachon University, Yeonsu-gu, Incheon, Republic of Korea

**Keywords:** glioblastoma, tumor antigen, vaccine platform, vaccine efficacy, vaccine perspective

## Abstract

Glioblastomas (GBMs) originate from glial cells and are characterized by aggressive growth and poor prognosis. Despite advances in surgical resection, complete elimination remains challenging, often leading to recurrence that is resistant to standard therapies. Immunotherapy and conventional treatments show promise in enhancing therapeutic outcomes across various cancers. Researchers continue to explore new treatments, particularly radiation, chemotherapy, and surgery; however, glioblastoma remains highly challenging, with only modest improvements in survival. Recent progress in immunotherapy, especially with tumor vaccines such as peptide-based and cell-based options (eg, dendritic cell vaccines), represents significant advancements despite the limitations observed in current clinical trials. This article reviews recent developments in vaccine-based immunotherapy for glioblastoma treatment.

## Graphical Abstract

**Graphical Abstract:**
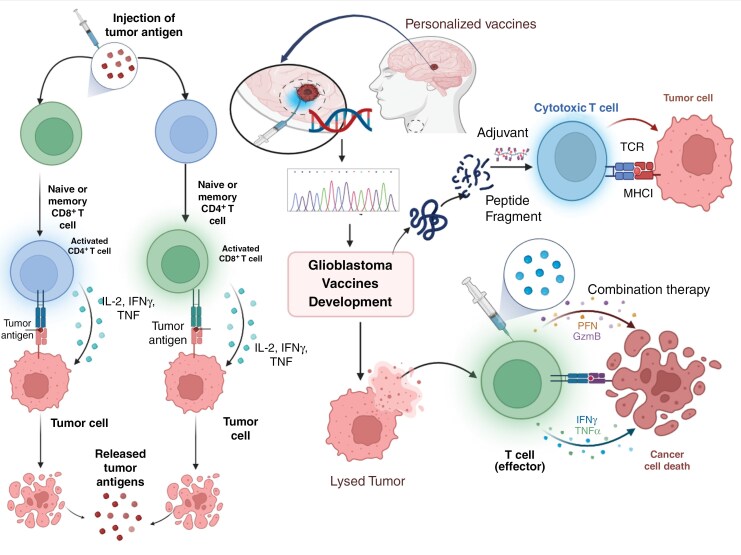

**Key Points**

1. GBM exhibits lethal progression, posing significant challenges for treatment.
2. Immunotherapy with conventional treatments shows promise in GBM treatment.
3. Tumor vaccine advances show promise, but efficacy assessment challenges persist.

Glioblastoma (GBM) develops through the proliferation of cells in the brain or spinal cord. It grows rapidly and has the potential to infiltrate and destroy normal brain tissue.^1^ Glioblastoma originates from astrocytes, which are a type of cell that nourishes nerve cells.^2^ Common GBM symptoms include headaches, vomiting, nausea, epileptic attacks, discrepancies in eyesight or speech, and cognitive problems.^3^ Nevertheless, the symptoms may differ depending on the location and size of the tumor.^4^ Glioblastoma is frequently treated with surgery, radiation, or chemotherapy.^5^ However, given the aggressive nature and potential for recurrence of glioblastoma, the prognosis for glioblastoma patients is frequently poor. Despite advancements in treatment, the median survival time for patients with glioblastoma is typically less than two years. Researchers continue to explore new treatment approaches and therapies, including targeted therapies, to improve outcomes for patients with glioblastoma. There are several modes of immunotherapy.^5^ However, managing glioblastoma remains a significant challenge in oncology. Adjuvant radiation, temozolomide (TMZ) chemotherapy, and tumor-treating fields (TTFs) are all standard of care (SOC) treatments for GBM. Immunotherapy to increase host immunity has long been considered a viable treatment strategy for GBM.^6^ The main variation between these antigen dependencies is whether a known antigen or group of known antigens is being targeted rather than whether tumor antigens are necessary. Antigen-independent treatments frequently seek to inhibit the immunosuppressive process of the tumor microenvironment (TME) to help T cells avoid fatigue, mount an antitumor immune response, or promote the release of tumor antigens.^7–15^ Neoadjuvant ICB immunotherapy has been shown by some researchers to improve overall survival (OS) in patients with recurrent GBM; however, no discernible alteration in immune checkpoint expression or cytotoxic T-cell activity has been observed in other studies, which may account for the inadequate survival benefit observed in GBM patients.^16–22^ The blood‒brain barrier (BBB), tumor heterogeneity, and glioma immunosuppression are among the limitations that impact the effectiveness of current treatment plans. Moreover, by increasing antigen exposure, oncolytic virus pretreatment can successfully increase the effectiveness of ICB therapy. There is an exploration of novel therapeutic options and some progress, particularly in vaccine therapy and tumor immunotherapy.^23–29^ The basic principle of vaccine treatment is the immune response specific to the injected exogenous antigens in the tumor.^23,30–33^ Therefore, introducing antigen-specific immunotherapy to a patient’s course of treatment could improve its clinical results. We address antigen-specific immune therapies for brain tumors in general in this review, but we pay particular attention to therapeutic vaccine platforms and types of GBM immunization. We also discuss the challenges, ongoing strategies, and possible ways to improve GBM immunotherapy in clinical practice.

## Mechanisms Implicated in the Diagnosis and Treatment of GBM

The structure of the brain is extremely complex and consists of several distinct regions, each with its function, such as the cerebrum, cerebellum, brainstem, thalamus, hypothalamus, amygdala, hippocampus, and cerebral cortex.^34^ These are just a few of the key structures of the brain, and each plays an important role in overall brain function. The brain is extremely flexible and malleable, which means that it may reorganize itself in response to learning, damage, and other events. Glioblastoma is an aggressive brain tumor caused by astrocytes, which are brain-supporting cells. It is the most prevalent and severe malignant primary brain tumor in adults.^34–47^ These tumors are designated grade IV astrocytomas, indicating that they are very malignant. Glioblastomas are very aggressive tumors that spread quickly and invade nearby brain tissue, as shown in Supplementary Figure S1.^48–56^ They are notorious for their capacity to spread rapidly throughout the brain, making total surgical eradication difficult.^57^ Overall, the diagnosis and treatment of glioblastoma are poor, necessitating a multimodal approach to therapy. Despite modern breakthroughs, it is still one of the most challenging malignancies to cure.

**Figure 1.**
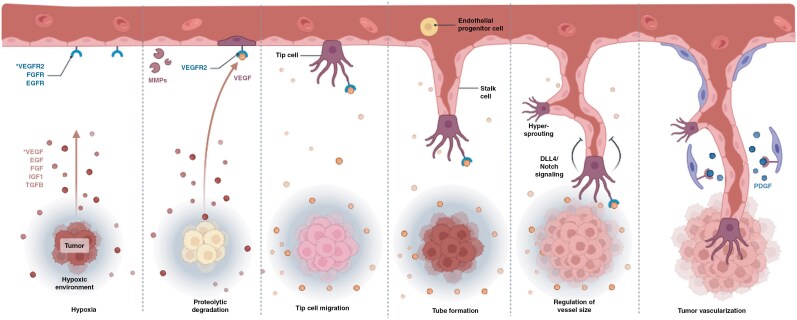
Angiogenesis is important in cancer development because solid tumors require a blood supply to grow beyond a few millimeters. Tumors can cause this blood supply to grow by releasing chemical signals that promote angiogenesis. Primary and metastatic brain tumors grow owing to their capacity to recruit blood vessels by coopting host vessels (cooption), sprouting new arteries (angiogenesis), and/or recruiting bone marrow-derived cells (vasculogenesis). The figure is created using Biorender.com with the following link. https://app.biorender.com/illustrations/621f35883514b0004c6ace28

The ECM influences brain tumorigenesis, particularly in the context of glioblastoma, including the tumor microenvironment, cell adhesion and migration, angiogenesis, and the modulation of signaling pathways^57^ (Figure 1). Endothelial junctions are specialized structures that form adjacent endothelial cells lining blood vessels, which are essential for the integrity and function of the BBB in the brain^58^ (Figure 1). The BBB is a highly selective barrier that regulates the passage of chemicals from the circulation to the brain parenchyma, protecting the brain from potentially dangerous compounds while allowing the required nutrients to enter, as shown in Supplementary Figure S1.^59^ Several variables contribute to angiogenesis in GBM, the most notable of which is vascular endothelial growth factor (VEGF). GBM cells thrive under hypoxic conditions and upregulate VEGF to promote angiogenesis.^60^ VEGF stimulates endothelial cell proliferation and vessel creation, hence facilitating tumor growth. Anti-angiogenic medicines, such as bevacizumab (a VEGF inhibitor), seek to starve the tumor by blocking its blood supply.^61^ However, these medicines frequently encounter difficulties, such as tumor adaptation and resistance. Angiogenesis targeting may improve the effectiveness of other therapies, such as chemotherapy and radiation, by normalizing the tumor vasculature.^62^ Liquid biopsies are a minimally invasive diagnostic method that examines tumor-derived components in bodily fluids, including blood, cerebrospinal fluid (CSF), or urine. Liquid biopsies can detect GBM early, assess therapy response, and explain tumor progression.^63^ Ongoing research is needed to validate liquid biopsy markers and incorporate them into standard GBM treatment. Liquid biopsies can detect biomarkers linked to angiogenesis (such as VEGF levels or endothelial-derived exosomes).^64^ Monitoring angiogenesis-related changes via liquid biopsy can help guide antiangiogenic therapy and identify resistance mechanisms. Researchers and physicians hope to improve glioblastoma diagnosis, monitoring, and treatment by inhibiting angiogenesis and utilizing liquid biopsy technology, resulting in better patient outcomes.^65^

Identifying the role of claudins in brain cancer may aid in the development of innovative treatment techniques that target the BBB or tumor cell behavior. Dysregulation of Claudine may increase BBB permeability, allowing cancer cells to infiltrate brain tissue and facilitating tumor development and progression.^66^ Claudins are essential components of tight junctions between cells, particularly those in the BBB.^67^ Their ability to regulate paracellular transport across epithelial and endothelial barriers is critical for tissue integrity and homeostasis, particularly in the brain. Claudins have sparked interest in the treatment of brain cancer, particularly glioblastoma multiforme (GBM), for a variety of reasons, including BBB integrity, tumor cell behavior, therapeutic targeting, and personalized medicine.^67^ However, further study is needed to determine the specific pathways by which claudins lead to brain cancer development and progression.^68^

Endothelial junction modifications and the inability of the BBB are prevalent characteristics of brain tumors, particularly glioblastoma. Endothelial junctions play a role in brain cancer by affecting the BBB, tumor angiogenesis, invasion, and metastasis, making them effective therapeutic targets.^69^ Identifying the role of endothelial junctions in brain cancer progression and BBB homeostasis is essential for developing successful therapeutic approaches. Studies designed to elucidate the molecular mechanisms underlying endothelial junction dysfunction in GBM may lead to the development of new treatments to improve the lives of patients.^70^ Angiogenesis, or the development of new blood vessels from existing vessels, is critical to the growth and spread of brain tumors such as glioblastoma. Angiogenesis contributes to brain tumor growth through nutrition and oxygen delivery; BBB disruption; invasion and metastasis; angiogenic factors; and therapeutic targets, as shown in Supplementary Figure S2.^70^

Liquid biopsy is a novel and effective technique for tracking tumor development and response to therapy in a variety of malignancies, including glioblastoma.^71^ Liquid biopsy is the process of analyzing blood or other bodily fluids for tumor-related biomarkers such as circulating tumor DNA (ctDNA), circulating tumor cells (CTCs), and extracellular vesicles. In the case of glioblastoma, liquid biopsy has several potential advantages, including noninvasive monitoring, early diagnosis of recurrence, assessment of therapy response, and discovery of therapeutic targets.^72^

**Figure 2.**
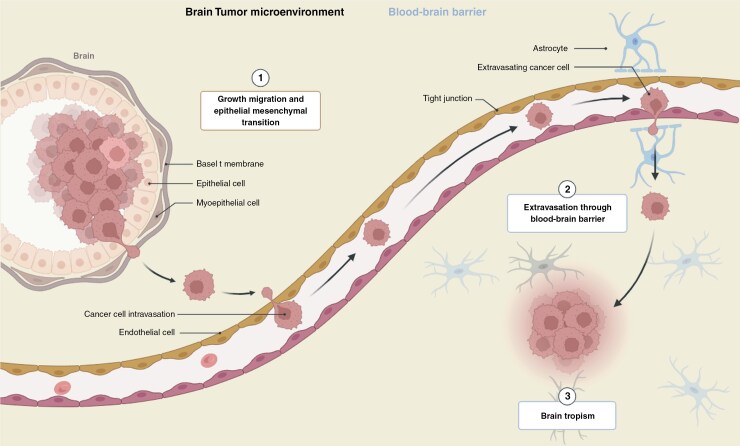
Microenvironmental Control of Tumor Progression and Therapeutic Response in Brain Metastasis. Cellular and noncellular components of the tumor microenvironment (TME) are important regulators of initial tumor growth, organ-specific metastasis, and treatment response. As a result, ctDNA has the potential to be employed for assessing tumor development and prognosis. In conclusion, liquid biopsy based on ctDNA analysis may constitute the next generation of tumor diagnostic and prognostic testing because of its excellent accuracy and sensitivity. The figure is created using Biorender.com with the following link. https://app.biorender.com/signup/payments?src=General%20proactive%20upgrade%20modal

Liquid biopsy strategies include the detection and monitoring of circulating tumor cells, cell-free DNA, and extracellular vesicles. Blood and urine are frequent samples used in liquid biopsy treatments.^73^ As a result, liquid biopsies are less intrusive to patients than tissue samples are, making them suitable for long-term monitoring of tumor growth. Liquid biopsy can detect a variety of molecular markers, including circulating cancer cells (CTCs), circulating tumor DNA (ctDNA), tumor-derived extracellular vesicles (EVs), tumor-educated platelets (TEPs), and circulating free RNA (cfRNA).^74^ Future studies are needed to answer biological questions about which cancer types can benefit from a liquid biopsy-based assay on the basis of known etiology, type, and degree of cfDNA or ctDNA foliation, as well as the mechanism behind foliation. In addition to previously documented methods of ctDNA release (apoptosis and necrosis), active secretion has been linked to various patterns of ctDNA fragmentation.^75^ Histone proteins form the nucleosome core, which protects ctDNA from nuclease cleavage. However, the remaining linker ctDNA sequence found between nucleosomes is extremely susceptible. Future research is needed to answer biological questions about which cancer types can benefit from a liquid biopsy-based assay on the basis of known etiology, the type and degree of cfDNA or ctDNA foliation, and the mechanism behind foliation. In addition to the previously described types of ctDNA release (apoptosis and necrosis), active secretion has been linked to different patterns of ctDNA fragmentation.^76^ Histone proteins construct the nucleosome core, shielding ctDNA from nuclease breakage. However, the remaining linker ctDNA sequence found between nucleosomes is particularly vulnerable. Overall, liquid biopsy is an effective method for tracking glioblastoma progression and guiding therapy options.^76^ More research and technological advances in this sector are needed to fully realize its potential for improving outcomes for individuals with this aggressive brain cancer, as shown in Figure 2.

**Figure 3.**
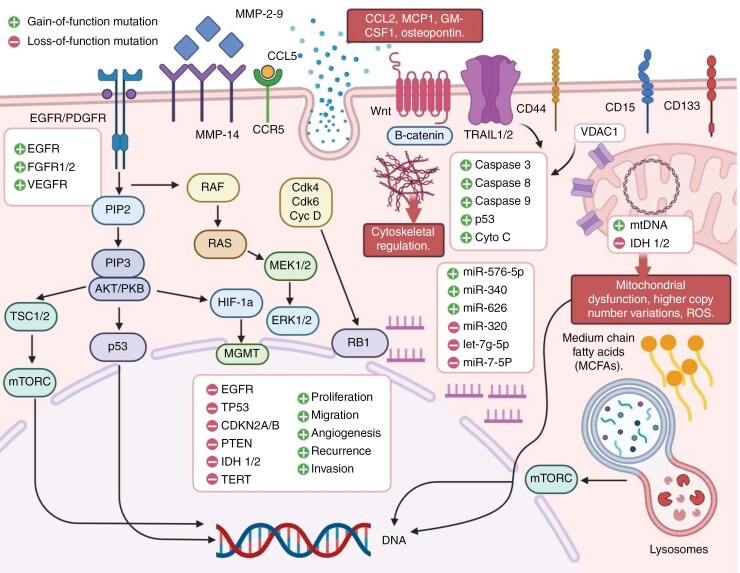
Different molecular mechanisms are implicated in the malignancy of GBM. The molecular mechanisms underlying GB are complicated, emphasizing the necessity for targeted treatment techniques. The deregulation of several molecular signaling pathways, the presence of the BBB, which prevents almost all chemotherapeutic agents from reaching the tumor site, and the presence of a population of stem-like cells known to be responsible for tumor recurrence after therapy can contribute to GB chemoresistance. GBM: Glioblastoma; MMP: Metalloproteinases; EGFR: Epidermal growth factor receptor; VEGFR: Vascular endothelial growth factor receptor; HIF-1a: Hypoxia-inducible factor 1a; miR: microRNA; cyto C: cytochrome C; mtDNA: Mitochondrial DNA; MCFAs: Medium-chain fatty acids; VDAC1: Voltage-dependent anion channel 1; RB1: Retinoblastoma. The figure is created using Microsoft PowerPoint.

GBM is the most severe and prevalent initial malignant brain tumor in adults. GBM is defined through multiple genetic variations, including mutations, amplifications, and deletions. The most common genetic abnormalities include loss of function of the tumor suppressor genes PTEN, TP53, and RB1, as well as overexpression of the oncogene epidermal growth factor receptor (EGFR), as shown in Figure 3.^77^ These alterations disrupt critical signaling pathways involved in cell cycle regulation, apoptosis, and DNA repair, resulting in uncontrolled cell proliferation and survival. Epigenetic changes, such as DNA methylation, histone modifications, and altered microRNA expression, play significant roles in GBM pathogenesis.^78^ These alterations can cause the silencing of tumor suppressor genes or the activation of oncogenes, both of which contribute to cancer formation and progression.^68,79–91^

GBM is characterized by numerous genetic alterations, including mutations, amplifications, and deletions. The most common genetic alteration is the loss of function of the tumor suppressor genes PTEN (phosphatase and tensin homologue), TP53 (tumor protein P53), and RB1 (RB transcriptional corepressor 1), as well as amplification of the oncogene EGFR, as shown in Figure 3).^92–101^ Tumor-associated macrophages and microglia (TAMs), as well as other immune cells, can promote tumor growth and invasion by secreting cytokines and growth factors that create an immunosuppressive environment and stimulate angiogenesis.^102–114^ The complex and heterogeneous nature of GBM presents significant challenges for effective treatment strategies.^69,87,115–131^ There have been several advancements in treatment approaches, particularly in vaccine therapy and tumor immunotherapy. The foundation of vaccine treatment is the immune response specific to the injected exogenous antigens in the tumor.^11,69,132–143^ The host immune response is induced and strengthened when foreign antigens are introduced to antigen-presenting cells. The main vaccines being tested in clinical trials for GBM are peptide-based vaccines and phytophthora seedling cells. This study examined the potential use of vaccination treatments to treat GBM.^144^

GBM patients with strong functional status (eg, Karnofsky performance status ≥ 70) often receive multimodal treatment. This comprises maximally safe surgical resection, chemoradiation with temozolomide (TMZ), and adjuvant TMZ therapy.^145^ Maximal safe surgery involves removing as much of the tumor as possible while preserving neurological function.^146^ A postsurgical MRI (within 24–72 h) was used to determine the degree of resection. Radiotherapy with doses of 60 Gray in 30 fractions (2 Gy per fraction) was administered over 6 weeks (5 days per week).^147^ Targets include the tumor bed and a margin to accommodate microscopic illness. Temozolomide (TMZ) should be taken orally at 75 mg/m² daily for the duration of radiation (7 days/week).^148^ TMZ is an alkylating agent that exposes tumor cells to radiation and causes DNA damage. Adjuvant temozolomide therapy is often initiated 4 weeks after chemoradiation to allow for recovery from acute toxicities. The dose schedule includes TMZ at 150 mg/m² daily for 5 days in a 28-day cycle.^149^ If tolerated, the dose was increased to 200 mg/m² daily for 5 days every 28-day cycle. 6 to 12 cycles, depending on the patient’s tolerance and clinical response. Supplemental therapy includes prophylaxis with ondansetron or similar medicines to treat TMZ-induced nausea, regular blood count monitoring (to identify neutropenia or thrombocytopenia), and liver function testing.^150^ Owing to the risk of lymphopenia, Pneumocystis jirovecii pneumonia prophylaxis is recommended during chemotherapy. Corticosteroids (eg, dexamethasone) are advised for side effect management to control cerebral edema, which decreases in severity as it is tolerated.^151^ To predict the TMZ reaction, molecular aspects must be considered. Patients with MGMT promoter methylation have improved outcomes. Although uncommon in GBM, IDH mutation-positive tumors have a better prognosis and may impact treatment options.^152^ Follow-up and monitoring can be accomplished via MRI surveillance every 2–3 months during adjuvant therapy and thereafter to monitor for recurrence. Repeat surgery, reirradiation, or second-line systemic treatments (such as bevacizumab and tumor-treating fields) can also be used to manage progression.^153^

Glioblastoma multiforme (GBM) is notoriously difficult to treat owing to its invasive growth style.^154^ GBM cells invade normal brain tissue, making surgical excision almost impossible. Even when a tumor appears to be completely gone on imaging, microscopic cancer cells are often left behind, leading to recurrence.^155^ Other variables that contribute to GBM incurability include the BBB. The BBB restricts the passage of many therapeutic agents to the brain, making it difficult for medications to efficiently target tumor cells.^156^ Glioblastoma multiforme (GBM) is notoriously difficult to treat because of its invasive growth pattern. GBM cells penetrate normal brain tissue, making surgical removal very difficult. Even when a tumor appears to be entirely gone on imaging, microscopic cancer cells are frequently left behind, resulting in recurrence. Other factors that contribute to GBM incurability include the BBB.^157^ The BBB inhibits the entry of many therapeutic drugs into the brain, making it difficult for treatments to effectively target cancer cells.

## Vaccine Platforms

GBM vaccination is a novel strategy to combat this aggressive and treatment-resistant brain malignancy. These vaccines are intended to activate the immune system to recognize and fight tumor cells.^158^ The following is a comparison of various GBM vaccination platforms, outlining their distinctions, benefits, and drawbacks. Each vaccine platform has unique benefits and drawbacks.^144^ The tumor antigen profile, patient-specific traits, cost, and logistics all influence the platform selection process. Advances in personalized medicine and combination therapy show potential for increasing GBM vaccination efficacy and patient outcomes.^159^ A comparison of various glioblastoma vaccine platforms, highlighting their differences, advantages, and disadvantages, is shown in Table 1. An antigen, a warning signal, and a method of delivering the antigen are the fundamental components of every vaccine design.^30^ These three factors interact to affect the effectiveness and spectrum of the vaccine. Clinical trials of tumor vaccine therapy for glioblastoma are described in Table 2.^144^ Next, we discuss the vaccine development platforms that incorporate these components and have been employed in GBM clinical trials, including peptide vaccines, mRNA vaccines, viral vector vaccines, and dendritic cell (DC) vaccines.^144^

**Table 1.** Comparing Various Glioblastoma Vaccine Platforms, Highlighting their Differences, Advantages, and Disadvantages

Vaccine platform	Description	Advantages	Disadvantages	References
Peptide-Based Vaccines	Utilize tumor-associated antigens (eg, EGFRvIII, survivin) in the form of synthetic peptides.	- Specific targeting of tumor antigens- Relatively easy to manufacture- good safety profile	- Limited to patients with specific tumor mutations- Potential for antigen escape- Limited immunogenicity	^160^
Dendritic Cell Vaccines	Use patient-derived dendritic cells loaded with tumor antigens to stimulate T-cell response.	- Personalized approach- Potent activation of T cells- Can use multiple antigens	- Complex and expensive manufacturing- Variable efficacy depending on the patient- Requires invasive procedures	^161^
Tumor Lysate Vaccines	Derived from whole tumor lysates containing a broad range of tumor antigens.	- Broad antigen coverage- Avoids the need to identify specific mutations- Suitable for heterogeneous tumors	- Risk of tolerizing immune response- Limited standardization- May include non-tumor antigens	^162^
DNA/RNA Vaccines	Deliver tumor antigen-encoding DNA or RNA directly into the patient to induce an immune response.	- Scalable and stable production- Can encode multiple antigens- Induces both humoral and cellular immunity	- Risk of low transfection efficiency- May require adjuvants for enhanced efficacy	^163^
Oncolytic Virus-Based Vaccines	Use engineered viruses to selectively infect and kill tumor cells while stimulating immunity.	- Combines direct tumor killing and immune activation- Can carry tumor antigens- Intratumoral delivery	- Risk of off-target effects- preexisting immunity to the virus- Complex regulatory requirements	^164^
Neoantigen Vaccines	Use patient-specific tumor neoantigens identified through genomic sequencing.	- Highly personalized- Targets truly tumor-specific antigens- Reduced risk of off-target effects	- High cost and time-intensive production- Requires advanced bioinformatics- Limited to patients with sufficient neoantigens	^165^
Heat Shock Protein Vaccines	Use tumor antigens chaperoned by heat shock proteins (HSPs) to enhance immune presentation.	- Efficient antigen presentation- Can carry multiple antigens- Potential for broad immune response	- Manufacturing challenges- May induce unwanted immune responses	^166^

**Table 2.** Clinical Trials of Tumor Vaccine Therapy for Recurrent Glioblastoma

Vaccine type	Vaccine and treatment	Tumor types	Enrollment	Study design	Phase of clinical tria	Main conclusion	Trial id	Reference
Survivin vaccine	SurVaxM + Pembrolizumab	rGBM	40	Double-blind Two arms	II	Ongoing	NCT04013672	^136^
HSP vaccine	HSPPC-96 + Bevacizumab	Surgically resectable rGBM	90	Open label Double-blind Three arms	II/III	Ongoing	NCT01814813	^137^
Multipeptide vaccine	HLA-A24–restricted vaccine candidates (ITK-1)	Recurrent or progressive supratentorial GBM	12	Open-label Dose escalation Cohort expansion	I	No serious adverse drug reactions were observed.	UMIN000001243	^30^
Multipeptide vaccine	IMA950/Poly ICLC + Pembrolizumab	Relapsing GBM	24	Open-label Dose escalation Two arms	I/II	Ongoing	NCT03665545	^138^
Multipeptide vaccine	EO2041 + Nivolumab + Bev acizumab	Progressive or first recurrent GBM	76	Open-label Dose escalation Three arms	Ib/IIa	All patients have been well tolerated through injections.	NCT04116658	^137^
Glioma-associated antigen vaccine	SL-701 + poly-ICLC	rGBM	74	Open-label Dose escalation Single arm	I/II	Ongoing	NCT02078648	^138^
Peptide vaccine	DSP-7888	Recurrent or advanced AML, MDS, GBM, melanoma,	24	Open-label Dose escalation	I	DSP-7888 Dosing Emulsion was well tolerated, with no dose-limiting toxicities.	NCT02498665	^139^
Peptide vaccine	DSP-7888	Recurrent or progressive supratentorial GBM	236	Open-label Double-blind Two arms	III	Ongoing	NCT03149003	^140^
Multipeptide vaccine	HLA-A*2402-restricted, modified 9-mer WT1 peptide vaccine	rGBM	21	Open-label Single arm	II	The overall response rate was 9.5%	NCT03149003	^136^
Multipeptide vaccine	PPV	HLA-A24–positive rGBM	88	Open-label Double-blind Two arms	III	No significant difference on median PFS	NCT04842513	^141^
DC vaccine	DCVax-L	Newly diagnosed GBM and rGBM	331	Open-label Double-blind Two arms	III	A group of 64 patients receiving only SOC plus placebo until recurrence	NCT00045968	^142^
Peptide-pulsed DC vaccine	αDC1 loaded with synthetic peptides + poly-ICLC	Recurrent malignant gliomas	22	Open-label Dose escalation Single-arm	I/II	The protocol was well-tolerated.	NCT00766753	^143^
Peptide-pulsed DC vaccine	Autologous DC vaccine + Bevacizumab	Newly diagnosed GBM and rGBM	35	Open-label Dose escalation Cohort expansion	I	25 patients diagnosed with rGBM were all well tolerated.	NCT02010606	^144^
Peptide-pulsed DC vaccine	GSC-DCV	Newly diagnosed GBM and rGBM	21	Open-label Dose escalation Two arms	II	10 patients with rGBM have a median OS of 10.7 months,	NCT01567202	^145^
Tumor lysate-pulsed DC vaccine	Gliadel Wafer + tumor lysate-pulsed DC vaccine	Primary and recurrent malignant glioma	28	Open-label Dose escalation Cohort expansion	I	The protocol was safe and elicited modest immunogenicity.	NCT01204684	^146^
Tumor lysate-pulsed DC vaccine	WT1-pulsed DC vaccine	Recurrent malignant glioma	10	Open-label Dose escalation	I	Vaccination therapy proved its safety, immunogenicity, and feasibility.	NCT 04615845	^147^
Tumor lysate-pulsed adjuvant DC vaccine	Adjuvant DC-based immunotherapy	Relapsed GBM	56	Open-label Three arms	II	The median OS and PFS was about 9.6 months	NCT04166006	^147^
Whole tumor vaccine	ERC1671 + bevacizumab	rGBM	9	Open-label Double-blind Two arms	II	The median OS of ERC1671 plus bevacizumab group was 12 months.	NCT01903330	^147^
EGFR-VIII peptide vaccine	Rindopepimut + TMZ	Relapsed EGFR vIII positive GBM	73	Open-label Double-blind Two arms	II	Statistically significant survival extension	NCT01498328 (ReACT trial)	^148^
Tumor lysate-pulsed DC vaccine	ATL-DC	Surgically accessible rGBM	40	Open-label Dose escalation Cohort expansion	I	Ongoing	NCT04201873	^149^
HSP vaccine	HSPPC-96	Recurrent resectable intracranial GBM	12	Open-label Dose escalation Cohort expansion	I	No adverse events attributable to the vaccine were found.	NCT02722512	^150^
HSP vaccine	HSPPC-96	Relapsed GBM	41	Open-label Dose escalation Single arm	I/II	Median overall survival was 42.6 weeks (95% CI 34.7–50.5).	NCT00293423	^150^

*rGBM- Reduced gradient bubble model (RGBM); HSPCC-Heat Shock Protein Peptide Complex-96; HLA-A24- human leukocyte antigen serotypeA 24; Poly ICLC- Polyinosinic-Polycytidylic Acid Stabilized with Polylysine and Carboxymethylcellulose;vDSP-7888 is a WT1-based cancer vaccine containing peptides that induce WT1-reactive cytotoxic T lymphocytes and helper T cells; AML-Acute myeloid leukemia; MDS -myelodysplastic syndrome; Human leukocyte antigens -HL;PPV- Pneumococcal Polysaccharide Vaccine; Ddendritic cell 1 -aDC1; A GSC-pulsed DC vaccine -GSC-DCV; Wilms’ tumor gene 1 -WT1; ERC 1671- Epitopoietic Research Corporation 1671; ATL-DC- Pembrolizumab and a Vaccine; HSP- Henoch-Schönlein purpur.*

## Peptide Vaccines

GBM is recognized for its large number of mutations because the protein and peptide changes produced by the mutant gene are unique to cancer cells and lacking in normal cells, it can be utilized as a specific antigen to elicit immune responses directed against tumor cells.^160^ These antigens are known as tumor-specific antigens (TSAs); however, they were formerly referred to as “neo-antigens.” Few mutations produce unique epitopes, and when these epitopes are presented by antigen-presenting cells in the human leukocyte antigen (also known as HLA), T cells respond.^160^ The high expression of epitopes and lack of selectivity in GBM make peptide vaccine-based techniques difficult to design. Investigators produced a peptide vaccination against a TSA in the late 1990s in an attempt to uncover and trigger immune responses to mutant sequences.^30^ CDX-110 (Rindopepimut) generates humoral and cytotoxic T-cell responses in mouse brain tumor models with high preclinical efficacy.^161^ Two recent studies highlighted the trend of tailored cancer vaccines against new antigens. In the first trial, a personalized cancer vaccine was created utilizing entire-exon sequencing data from a resected tumor and matching normal tissues to combat a new antigen. Each patient was given a vaccine comprising 7 to 20 antigens that have a high affinity for binding to HLA type I-human leukocytes type 1.^162^

Peptide vaccines are made more quickly and cheaply than are cell-based vaccines. These patients are immunized via chemically synthesized in vitro peptide sequences that are either unique to tumors or highly expressed in tumors.^163^ Therapeutic cancer peptide vaccines fall into two primary categories: synthetic long peptides (approximately 25–30 amino acids) and minimum peptide epitopes (approximately 8–11 AAs).^164^ Without going through an internalization phase, the minimum peptide epitope may connect directly to the I-binding groove of the major histocompatibility complex (MHC). It also increases the possibility of binding to non-APC-nucleated cells that express MHC I molecules but do not have costimulatory molecules.^164^ This might result in peptide tolerance or inadequate T-cell activation. SLPs need APC processing as opposed to minimum peptide epitopes. APCs may effectively present MHC I- and MHC II-restricted epitopes through cross-presentation due to their extended length. Following in vivo injection, autologous CD4 + and CD8 + T lymphocytes are prepared to cytotoxically circulate to the tumor after APCs ingest and deliver SLPs to them.^165^ Peptide vaccines require the inclusion of additional immune adjuvants because of their inability to stimulate the innate immune system. Adjuvant support guarantees that APCs provide enough costimulatory signals to trigger a strong T-cell response.^166^ Peptide vaccines exploiting tumor-associated antigens (TAAs), such as EGFRvIII, IDH1-R132H, or survivin, have been studied in various clinical trials. ACT IV (NCT01480479), which studies rindopepimut (an EGFRvIII-targeted vaccine) in newly diagnosed GBM, is one of the most notable and was eventually discontinued for lack of efficacy; however, earlier-phase studies in rGBM (such as the ReACT, NCT01498328) revealed a modest survival advantage with rindopepimut when combined with bevacizumab.^167^ Peptide vaccines remain a potentially viable option, particularly in combination with adjuvants or immune checkpoint inhibitors.

## Dendritic Cell Vaccines

The most competent APCs at inducing autoimmune reactions are DCs. DCs occur mostly during the immature stage before antigen absorption.^168^ By encouraging the release of different cytokines and the upregulation of surface MHC, costimulatory molecules, and cytokine receptors, antigen capture accelerates the maturation of DCs.^168^ Antitumor CD8 + T-cell responses are initiated in three consecutive steps: tumor antigen absorption and cross-presentation, tumor antigen-specific CD8 + T-cell priming by DCs, and tumor cell destruction by effector CTLs.^169^ It is essential to provide DC tumor antigens either ex vivo (DC vaccination) or in the form of peptide vaccines to stimulate the production of effector T lymphocytes specific to tumors. Peptide vaccines require the inclusion of additional immune adjuvants because of their inability to stimulate the innate immune system.^169^ Adjuvant support guarantees that APCs provide enough costimulatory signals to trigger a strong T-cell response. Peptide vaccines, such as SurVax (survivin) and Rindopepimut (EGFR III), are the most widely used GBM vaccines.^170^ DC vaccines are created by pulsing autologous dendritic cells with either tumor antigens or lysates. For example, the DCVax-L vaccine utilizes autologous tumor lysate-pulsed DCs. A phase III clinical trial (NCT00045968) with the DCVax-L vaccine in recurrent GBM revealed promising results, especially among the subgroup of recurrent GBM patients who achieved a prolonged overall survival benefit.^171^

## mRNA Vaccines

One strand of DNA is converted into a monomeric molecule called mRNA in the nucleus of cells. This endogenous mRNA is translated into proteins in the cytoplasm by ribosomes after transcription.^144^ By using this approach, researchers have generated vaccine-grade mRNAs. On the other hand, mRNA techniques induce immunogenic cell death by causing cancer cells to release tumor antigens directly. These techniques not only speed up the procedure but also make it possible to generate customized vaccinations for tumor removal.^172,173^ Since mRNA vaccines are customized, the immune system may more effectively target cancer cells while minimizing damage to healthy cells. However, the adaptability of mRNA vaccines is demonstrated by their compatibility with different cancer treatments. The use of mRNA vaccines for brain cancer is still in its early phases, despite promising results in other disease areas.^173^ DCs have been a major target in neoantigen and/or mRNA vaccine research for brain cancer because of their efficacy in stimulating immunity and the precedent of one FDA-approved immunotherapy (Provenge). It has been demonstrated that DC-based vaccinations are beneficial against malignancies, such as brain cancer and glioma.^174^ A recent phase III clinical trial examined the effectiveness of supplementing conventional glioblastoma therapy with DCVax-L, a tumor lysate-based DC vaccination. According to these findings, individuals with newly diagnosed and recurrent glioblastoma had a longer survival rate when DCVax-L was added to conventional therapy.^171,175–177^ This finding implies that DCVax-L may be a potential supplement to the available glioblastoma therapies. There have been few published studies on mRNA-based vaccines. Seven patients with glioblastoma received a DC vaccine containing cancer stem cell (CSC) mRNA in groundbreaking research.^171^

The accessibility of cell-surface proteins, their involvement in critical signaling networks, and their dysregulation in cancer make them particularly interesting targets. They have the potential to be employed in both chimeric antigen receptor (CAR)-based immunotherapy and mRNA vaccination procedures.^178^ The use of antigens (FCGBP, FLNC, TLR7, and CSF2RA) has aided in the development of mRNA cancer vaccines.^173^ Researchers also identified ARPC1B and HK3 as potential mRNA antigens for building a GBM mRNA vaccine via the same technology and analysis, and they determined that patients in IS2 were the best candidates for GBM immunization.^179^ MMP9 and SLC16A3 were identified as antigens for GBM, whereas PTBP1 and SLC39A1 were chosen as antigens for LGG. Four overexpressed and mutant tumor antigens, TP53, IDH1, C3, and TCF12, are associated with a poor prognosis and antigen-presenting cell infiltration in glioma patients.^180^ These four coldeoantigens are effective antigens for developing antiglioma mRNA vaccines.^181,182^ After examining RNA-seq data and clinical information from more than 1000 patients, four glioma antigens, ANXA5, FKBP10, MSN, and PYGL, which are associated with improved prognoses, were identified.^183^ Furthermore, they may act as potential antigens in generating an antiglioma mRNA vaccine, particularly for those with immunological subtypes 2 and 3. Ongoing investigational trials using mRNA vaccines (eg, those targeting IDH1 mutations or multiple TAAs) are currently being conducted for early-phase evaluation (eg, NCT03893903).^184^ The use of GBM-associated surface antigens to identify novel treatment targets for GBM via an mRNA approach has been revealed.

## Viral Vector Vaccines

Antigens have been delivered in clinical settings via several recombinant viral vectors.^185^ Viral pathogen-associated molecular patterns not only elicit a strong immune response via antigen delivery but also boost the recipient’s immune system.^186^ However, prolonged use of viral vectors may result in increased humoral immunity to viruses. Clinical experiments using viral vector vaccines to target the human cytomegalovirus peptide in GBM are still underway (NCT03382977).^186^

## Tumor Antigens in GBM

The effectiveness of tumor vaccination depends on the choice of antigens.^187^ An ideal cancer antigen should be highly immunogenic and present in practically all tumor cells.^188^ An efficient T-cell response can be induced only by nonself-antigens that are distinct from peptides prevalent in normal peripheral tissues. Moreover, for cancer cells to evade exogenous tumor antigen-induced immunosurveillance,^189^ antigen loss must occur throughout tumor growth for an optimal tumor antigen to be expressed widely and steadily.^189^ Not every peptide produced from tumors is antigenic. Owing to MHC limitations, only peptides with matching attach residues and a fitting length are permitted to be presented by MHC molecules.^190^ Stronger T-cell antitumor activity is correlated with a peptide that has a high affinity for MHC molecules. Similarly, MHC limitation affects the immunogenicity of peptides.^191^ Tumor cell intracellular proteins are the source of endogenous antigens, which include neoantigens (tumor-specific antigens, or TSAs) and TAAs.^162^ Recently, interest in the use of exogenous antigens in cancer vaccination treatments has increased. Cancer MHC presents these antigens, which are produced from cancer cells that preferentially infect pathogens and trigger the T-cell response. Notably, sources of bulk tumor-derived antigens from GBM, such as tumor lysate vaccines such as AV-GBM-1 and GBM6-AD95, or tumor-mRNA vaccines, may contain these antigen categories as well as as yet unrecognized antigen classes.^180^

Cancer-testis antigens (CTAs) are more abundant in cancer cells and germ cells (such as the testis) than in other organs, and they contribute to meiosis, aberrant chromosomal segregation, and aneuploidy.^191^ Tumor-overexpressed antigens are proteins that are significantly overexpressed in tumors relative to healthy tissues. These are the three types of TAAs in GBM.^192^ TAAs can prolong overall survival in GBM vaccination, as demonstrated by the promotion of CD8 + T-cell activation and cytotoxicity, which is facilitated by increased CTA expression in GBM cells.^193^ Neoantigens and TAAs produced successful immunotherapies, particularly for tumors with low TMB, according to a recent clinical study on newly diagnosed GBM patients.^194^

Identifying antigens for GBM vaccination requires advanced approaches aimed at identifying tumor-specific or TAAs capable of eliciting a strong immune response.^162^ These strategies concentrate on identifying antigens expressed exclusively or predominantly in tumor cells to reduce off-target effects and maximize therapeutic efficacy. Antigens for GBM vaccination are frequently identified via a mix of genomic, proteomic, and immunological approaches, as well as modern analytics. Advances in single-cell technology and personalized medicine are increasing the precision and application of GBM vaccinations.^195^ Antigens for glioblastoma vaccination are identified via a variety of techniques, each utilizing modern technologies to discover tumor-specific or tumor-associated targets, as shown in Table 3.

**Table 3.** The Identification of Antigens for Glioblastoma Vaccination Involves Multiple Methodologies

Method	Description	Key Features	Examples of Identified Antigens	References
Genomic Sequencing	High-throughput sequencing of tumor DNA to identify mutations, amplifications, or deletions.	- Detects tumor-specific neoantigens - Requires bioinformatics for analysis	EGFRvIII, IDH1 R132H mutations	^200^
Transcriptomics (RNA-Seq)	Sequencing of tumor RNA to identify overexpressed genes and novel transcripts.	- Identifies aberrantly expressed or fusion genes - Can infer protein-level antigen candidates	Survivin, SOX2	^201^
Proteomics	Mass spectrometry to analyze the tumor’s protein expression profile and identify unique peptides.	- Detects post-translational modifications - Validates antigen expression at the protein level	HER2, IL13Rα2	^8^
Immunopeptidomics	Mass spectrometry analysis of peptides bound to MHC molecules on tumor cells.	- Identifies directly presented antigens - Prioritizes epitopes recognized by T cells	Tumor-associated neoantigens presented by MHC class I/II	^202^
Bioinformatics and Machine Learning	Computational prediction of potential neoantigens based on sequencing data.	- Predicts binding to MHC molecules - Accelerates antigen discovery	Predicted neoantigens specific to individual tumors	^203^
Serological Analysis	Identification of antigens recognized by autoantibodies in patient serum.	- Reflects antigens naturally targeted by the immune system	NY-ESO-1, MAGE-A1	^204^
In Situ Hybridization	Localization of mRNA expression in tumor tissues to confirm tumor-specific gene expression.	- Validates spatial distribution of antigen expression - Complements RNA-Seq and proteomics	EGFRvIII expression in tumor cells	^205^
Reverse-Phase Protein Arrays (RPPA)	High-throughput detection of protein expression in tumors.	- Quantifies protein levels - Suitable for large patient cohorts	p53, VEGF	^206^
Functional Screens	CRISPR/Cas9 or RNAi screens to identify essential tumor antigens or immune targets.	- Focuses on antigens critical for tumor survival - Links function to antigenicity	Targets identified through synthetic lethality	^207^
Tumor Lysate or Exosome Analysis	Use of tumor-derived lysates or exosomes to identify immunogenic proteins.	- Enriches for tumor-restricted antigens - Includes a wide array of potential targets	Heat shock proteins, unique exosome-associated antigens	^208^

## Immunosuppressive TME in GBM

GBM is recognized as having an immunosuppressive microenvironment that protects tumor cells from immune attack.^196^ This tumor persists by recruiting and reprogramming immune cells and establishing a so-called “cold” tumor where there is no significant immune infiltration/activation.^196^ GBM is associated with high infiltration of immunosuppressive cells, such as tumor-associated macrophages (TAMs), regulatory T cells (Tregs), and myeloid-derived suppressor cells (MDSCs).^189^ More knowledge on the immunological landscape of GBM will therefore be important because of the profoundly immunosuppressive tumor microenvironment (marked by inadequate immune cell infiltration, T-cell exhaustion, and the composition of suppressive immune cell populations) and the complex immune landscape that facilitates tumor growth and can restrict the potential for efficacy of immune-based therapeutic strategies.^144^ As such, recognizing aspects of immune evasion applied by GBM is necessary and relevant to developing effective immunotherapeutic approaches specific to the tumor and microenvironment.^197^ The tumor enlists and reprograms different immune cells, effectively creating a “cold” tumor that cannot recruit and activate immune cells effectively. This occurs despite high infiltration of immunosuppressive immune cells (eg, TAMs, regulatory T cells [Tregs], and MDSCs).^197^ Glioblastoma is uniquely resistant to immunotherapy, in part because of its highly immunosuppressive microenvironment and immune-privileged site in the brain.^197^ The field is currently working to better understand and overcome this barrier through combination strategies that exploit the immune system and induce responses in the tumor.^189^

The minimal burden of tumor mutations and TMB levels remain chronically low in CNS tumors, such as GBM, which may be due to epigenetic alterations.^196^ Few mutations can be found in GBM cells as neoepitopes for effective autologous T-cell identification, since neoantigen presentation is a probabilistic process that is dependent on neoantigen quantity.^198^ Antigen dissemination is an important immunotherapy response mechanism that occurs during therapeutic tumor vaccination. It is produced by increased exposure to tumor antigens, which is linked to early tumor lysis triggered by vaccination.^144^ The benefits of antigen dispersion after immunotherapy may be countered by a smaller antigen pool caused by low TMB, which exposes fewer immunogenic neoantigens following vaccination.^199^ Hypoxia-induced T-cell sequestration may negate the therapeutic effects of bevacizumab treatment. Effector T cells in GBM develop fatigue characteristics, which are linked to several TME-related variables.^200^ In GBM, IL-10 is essential for the development of an immunosuppressive TME because it suppresses APCs, impedes T-cell proliferation, and activates regulatory T (Treg) cells. It encourages Treg differentiation by inducing tolerogenic DC maturation.^189^ Research has demonstrated that individuals with gliomas have a relatively high percentage of CD4 + Treg cells in their blood and tumors. Neutralizing antibodies against CD25, which impair the suppressive activity of Tregs, can restore suppressed cytotoxic T-cell antitumour activities in mice.^201^ Furthermore, intratumoural regulatory T cells (Tregs) possess a unique TCR repertoire that identifies tumor neoantigens, or TAAs, indicating that the tumor antigens might result in the colony growth of tumor antigen-recognized regulatory T (Treg) cells. Reducing the number of Treg clones that recognize cancer antigens increases effector/memory T-cell antitumour responses against the same tumor antigens.^144^ TAMs, which are composed of MDSCs and TAMs, are another critical component in the formation of an immunosuppressive TME in GBM.^197^

Upregulated GMCSF expression in the GBM TME leads to increased IL-4Rα expression in GBM-infiltrated myeloid cells.^202^ This IL-13-induced synthesis of arginase inhibits T-cell proliferation and function. The suppression of T-cell activity can be considerably reversed by eliminating MDSCs from the peripheral circulation. A prior study revealed that MDSCs accumulate in the peripheral blood of GBM patients.^203^ Upregulated GMCSF expression in the GBM TME leads to increased IL-4Rα expression in GBM-infiltrated myeloid cells.^203^ This IL-13-induced synthesis of arginase inhibits T-cell proliferation and function. The suppression of T-cell activity can be considerably reversed by eliminating MDSCs from the peripheral circulation. Interaction with the vaccine for at least five days induces T-cell anergy mediated by IFN-γ and MDSCs.^204^ One of the resistant cancers with the highest degree of heterogeneity is GBM, particularly at the molecular level.^205^ Moreover, the immunoediting effect and the balance between immune surveillance and carcinogenesis continuously shape the pool of tumor antigens during postsurgical recurrence. The effectiveness of immunotherapy is limited during the escape phase by the predominance of immunosuppressive characteristics and the elimination of numerous immunogenic antigens.^206^

## Clinical Characteristic Challenges in GBM Patients

Tumor vaccines are more advantageous to patients in their early years.^207^ Changes in the natural immune system associated with aging may harm the efficiency of tumor vaccination.^208^ Age-associated involution of the thymus adversely impacts the immune response, resulting in reduced tumor immune surveillance.^209^ This leads to a reduction in the variety of the peripheral T-cell repertoire and immunosenescence, which impairs the ability to recognize and respond to novel stimuli.^210^ Age-related atrophy of deep cervical lymph nodes (DcLNs), which are thought to constitute the primary secondary lymph organ response to CNS tumors, further reduces the T-cell compartment.^211^ The function of the dural lymph vessels that drain CSF decreases with age. Dysmorphology, increased lymphatic channel thickness, and dural lymph vessel dysfunction are linked to aging.^212^ Age-related inflammatory conditions may also lessen the efficiency of vaccinations by further inhibiting antigen-specific immunity. Patients who achieved a complete response (CR) in a phase I/II study of oncolytic DNX2401 virotherapy with ICB in recurrent GBM patients were younger than 30 years, indicating that age may be a protective factor.^213^

The exact process of cancer antigen presentation from parenchymal to dc-LNs has been identified by researchers, with structural lymphatic vessels and the glymphatic system in the brain.^214^ The subarachnoid lymphatic-like membrane (SLYM) and the dural lymph vessel, which are located close to the main site and are used by APCs, especially DCs, for immune monitoring, may be irrevocably destroyed during treatment.^215^ Data indicating that the meningeal lymphatic vasculature affects both neuroinflammation and CNS lymphatic outflow support these findings. Moreover, patients’ postoperative inflammatory diseases may exacerbate their immunosuppression.^216^ More studies are needed to determine the exact process of brain tumor antigen outflow into the lymph nodes.

Various hematological and solid tissue tumors respond favorably to T-cell immunotherapy platforms. However, T-cell dysfunction significantly decreases the effectiveness of these approaches in the management of GBM. GBM exhibits inherent and therapy-driven clonal dynamics as the illness progresses.^217^ Multiple neoplastic treatments, including chemotherapy and radiation, as well as age-induced immunosenescence, promote thymocyte death, hasten thymic involution, and reduce lymphoid precursors. Clinicians commonly employ corticosteroids to control the course of peritumoral edema.^218^ In theory, a decrease in the number of corticosteroid-mediated immune cells might negate the anticancer benefits of cancer vaccines; however, no phase III clinical trial results have been published that definitively reveal a deleterious effect on GBM patient survival.^219^ Alternatives to steroids should be studied when developing GBM vaccines. Bevacizumab is effective at controlling cerebral edema. The combination of GBM vaccination therapy with bevacizumab is a prospective steroid treatment for edema.^220^

## Personalized Neoantigens

GBM patients differ from one another; hence, the majority of neoantigens produced by these mutations are rarely shared.^221^ Compared with priming T cells with all tumor lysate proteins in an unselective manner, personalized neoantigen creation via bioinformatic prediction has the potential to improve the clinical effectiveness of GBM patient vaccination. Moreover, by including numerous private cancer antigens, primed T cells recognize a significant fraction of tumor cells with distinct antigens, thereby lowering the tumor burden and mitigating the chance of antigen loss.^222^ CD4 + helper T cells are essential for the survival of CD8 + T-cell populations to recognize tumor antigens. After presenting tumor antigens, DCs activate CD4 + T cells, resulting in IL-2 production and T-cell proliferation and development.^223^ Prepared CD4 + T cells produce IL-21, which induces CD8 + T cells to establish a cytotoxic phenotype and prevents them from increasing into collapsed subclones.^224–227^ A recent study revealed a cellular triad associated with anticancer activities.^223^ The intratumoral cellular triad consists of DCs, CD4 + T cells, and CD8 + T cells. The presence of spatially attracted CD4 + T cells and DCs on the outskirts of CD8 + T cells boosts local CD8 + T-cell differentiation into effector T cells and increases CD8 + T-cell cytotoxicity.^228–231^ Antigen-induced antitumor responses require both CD4 + and CD8 + T cells that have been primed with tumor antigens. CD4 + T-cell priming ensures that tumors are effectively rejected after immunization.^232,233^ The degree of CD8 + T-cell infiltration alone is not a significant predictor in glioma patients, underscoring the importance of CD4 + T cells, which have been neglected in previous glioma antigen studies. GBM immunization increased both CD4 + and CD8 + T lymphocytes.^224^

Long-term vaccination increases IFN-γ levels, resulting in increased expression of immune checkpoint markers. To combat this immunosuppressive procedure, ICB is an effective complement to vaccines.^234,235^ Combination ICB therapy increases vaccine antitumor efficiency in both preclinical mouse cancer models and clinical trials and can even result in a rapid and long-lasting complete response in patients with metastatic tumors. The combined administration of ICB and neoantigen vaccines improved survival in an ICB-resistant GBM mouse model. Additional investigations using armed oncolytic viruses for GBM are also ongoing. This method allows the combination of tumor antigens with oncolytic viruses to enhance their anticancer properties.^235^ In addition, a multiphase combination treatment strategy may be a useful supplemental regimen after SOC. During chemotherapy and oncolytic virus treatment, the TME might switch from “cold” to “hot,” resulting in the release of tumor-lysed antigens.^9^ These tumor-lysed antigens are important sources of ICD-induced antigens. In addition to preloaded antigens, ICD-induced antigens will increase the efficacy of future DC vaccination therapies.

## Future Prospective

Tumor vaccines are an effective approach for improving the therapeutic impact of SOC on GBM patients. GBM is a malignant tumor that may have originated from an aberrant epigenome. It has a low TMB and few neoantigens, which may impair immunotherapy effectiveness. Several clinical studies of GBM vaccinations have reported disappointing results, with limited therapeutic advantages.^144^ Future areas for improving GBM vaccine efficacy will include vaccination design optimization, such as tailored antigen selection, multiantigen targeting, and vaccine platform or adjuvant development. Another challenge is developing a strong antitumor response in patients to overcome the immunosuppressive properties of GBM.^236^ Tumor vaccines are an excellent way to improve the treatment efficacy for GBM patients after SOC. GBM is a malignant tumor that can develop from an abnormal epigenome. It has a low TMB and few neoantigens, which might reduce immunotherapy effectiveness. Several clinical studies of GBM vaccinations have reported inadequate outcomes and no therapeutic effects. Another challenge is creating an effective antitumor response in patients to counteract the immunosuppressive effects of GBM. In the context of cancer vaccine therapy, investigating how it can improve a GBM patient’s immune system, which has been impaired by age, medicine, or the tumor immune escape mechanism, is vital.^144^ Tumor vaccination is effective when combined with other immunotherapies, such as ICB. Combination therapy and immunization of premalignant LGG patients provide innovative approaches for improving vaccination regimen design.^236^ While each vaccination platform (mRNA vs. peptide, DC loading vs. viral vector) and antigen source has recognized and claimed benefits and drawbacks, current and future research must investigate the differences between them.

## Concluding Remarks

The GBM tumor vaccine shows potential as a supplement to SOC. Personalized multipeptide vaccinations containing neoantigens and TAAs outperform single-peptide vaccines in terms of cancer regression and antitumor responses. Owing to the unique epigenetic process of gliomagenesis, the number of neoantigens available for GBM immunotherapy is expected to be restricted, and TAAs and pathogen-derived antigens may be effective in expanding the vaccine-antigen arsenal. The efficacy of GBM vaccines can be influenced by patient age, immunological fitness, and treatment regimens. Future therapeutic applications of GBM tumor vaccines will focus on the most efficient combination of cancer vaccinations and SOC. Optimizing vaccine design (antigen and adjuvant selection), concentrating on the role of CD4 + T cells in tumor vaccines, and utilizing combination immunotherapy will be new keys to increasing the efficacy of GBM vaccines.

## Supplementary Material

Supplementary material is available online at *Neuro-Oncology Advances* (https://academic.oup.com/noa).

vdaf135_suppl_Supplementary_Figures
