# Supplementary material for: Advancing vaccine-based immunotherapy in glioblastoma treatment
Source: Neurooncol Adv. 2025 Jun 24;7(1):vdaf135. doi: 10.1093/noajnl/vdaf135 (PMC12314604; doi:10.1093/noajnl/vdaf135)
Supplement: vdaf135_suppl_Supplementary_Figures [file vdaf135_suppl_supplementary_figures.docx]

**
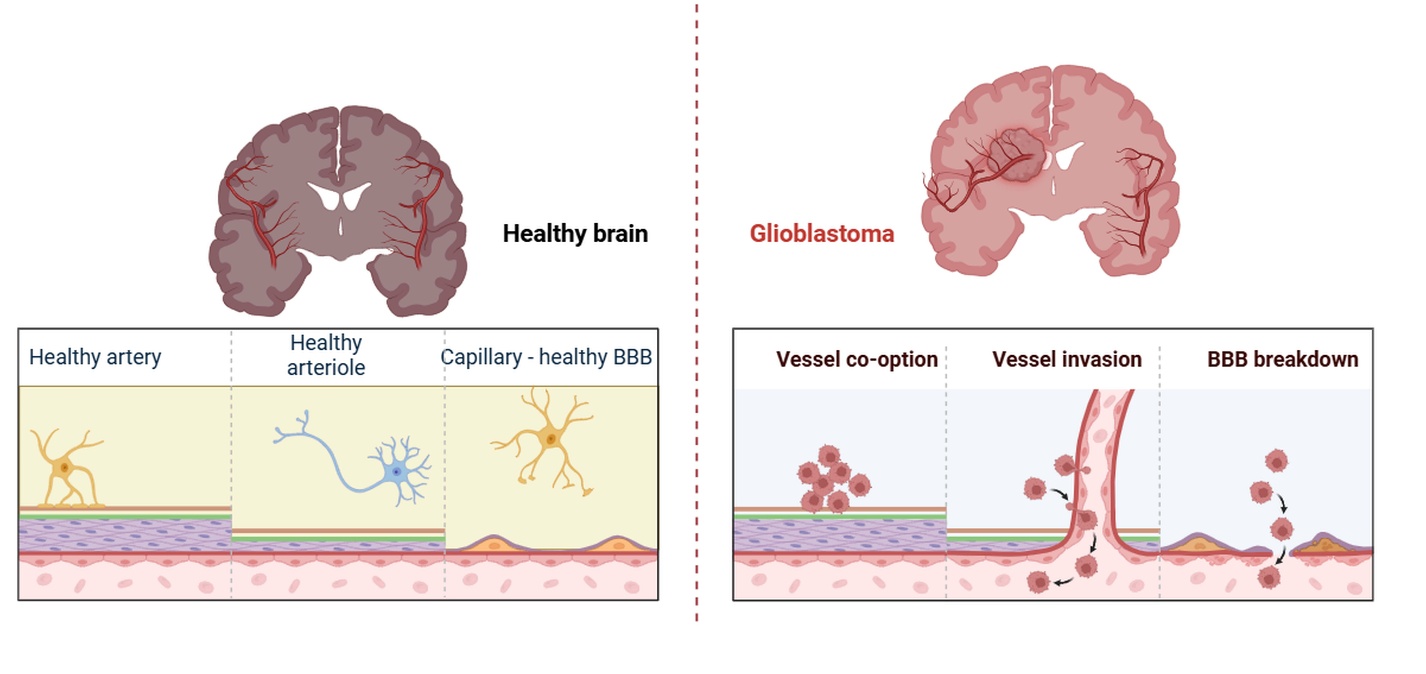
**

**Figure S1.** Healthy Brain vs. Glioblastoma A healthy brain represents the healthy artery, healthy arteriole, capillary, and healthy BBB, and a brain with glioblastoma represents vessel co-option, vessel invasion, and the BBB. The figure is created using Biorender.com using the following link.

<https://app.biorender.com/signup/payments?src=General%20proactive%20upgrade%20modal>

**
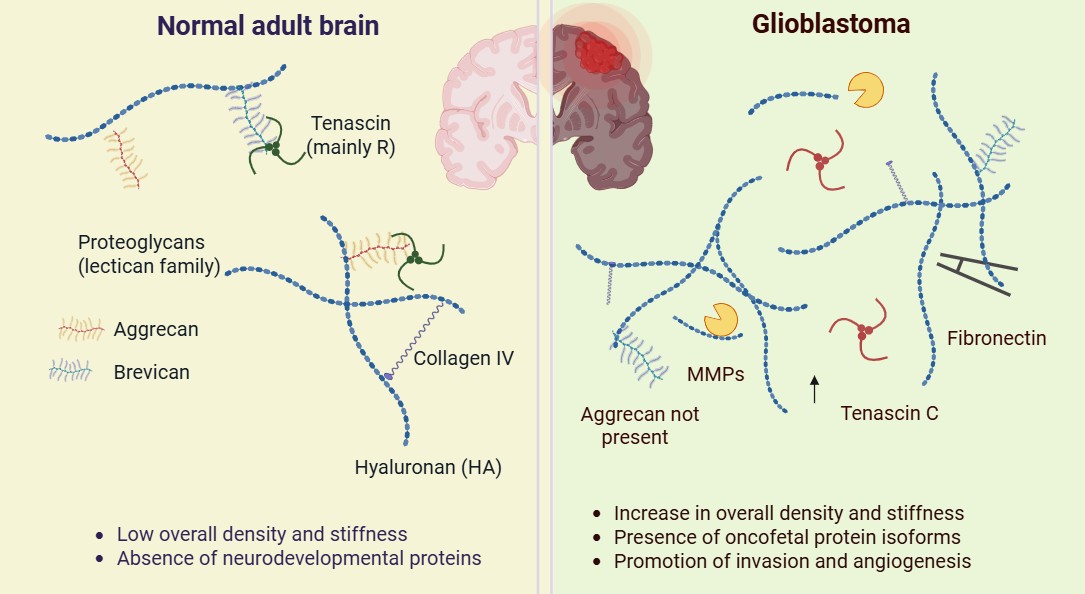
**

**Figure S2.** Extracellular Matrix in Brain Tumorigenesis:

- Normal adult brain: low overall density and stiffness; absence of neurodevelopment proteins.
- Glioblastoma: Increases in overall density and stiffness; presence of onco-fetal protein isoforms; Promotion of invasion and angiogenesis

The figure is created using Biorender.com using the following link.

<https://app.biorender.com/signup/payments?src=General%20proactive%20upgrade%20modal>
